# Efficacy and Safety of Glucocorticoid Monotherapy Versus the Combination of Glucocorticoid and Immunosuppressive Agents for Immunoglobulin G4-Related Disease: A Systematic Review and Meta-Analysis

**DOI:** 10.7759/cureus.47099

**Published:** 2023-10-16

**Authors:** Norah I Alsalamah, Bayader Alhrabi, Norah Alhumaily, Rawad AlHadidi, Lujainah S Basubrain, Zahra Al Asmari

**Affiliations:** 1 PharmD, King Saud Hospital Unaizah, Unayzah, SAU; 2 Pharmacy, Suliman Al-Habib Hospital, Riyadh, SAU; 3 PharmD, Al-Maali Hospital, Riyadh, SAU; 4 Pharmaceutical Care Department, Ministry of National Guard - Health Affairs, Jeddah, SAU; 5 PharmD, King Abdulaziz Medical City Riyadh, Riyadh, SAU

**Keywords:** a meta-analysis, a systematic review, immunoglobulin g4-related disease, immunosuppressive agents, glucocorticoid therapy

## Abstract

We conducted the current systematic review and meta-analysis to evaluate the efficacy and safety of the combination of glucocorticoid and immunosuppressive agents (IM) compared to glucocorticoid (GC) monotherapy for the treatment of immunoglobulin G4-related disease (IgG4-RD). PubMed, Web of Science, Scopus, OVID, and the Cochrane Library were searched for related articles. Meta-analysis was conducted with outcomes including relapse rate, remission, and adverse events. We calculated the odds ratio (ORs) and 95% confidence interval (CI) with the meta-analysis model. Ten studies involving 906 patients were included in the systematic review; of them, seven studies were included in the meta-analysis. The effect size showed that the GC group was associated with a higher relapse rate (OR = 2.97, 95% CI [1.91, 4.62], p < 0.0001) and a less complete remission rate (OR = 0.27, 95% CI [0.16, 0.47], p < 0.0001) than the combination of GC and IM group. While there was no significant difference between the two compared groups in terms of adverse events (OR = 0.73, 95% CI [0.44, 1.21], p = 0.22). No significant heterogeneity was detected regarding all outcomes (p > 0.1, I2 < 50%). Treatment of IgG4-RD patients with a combination of GC and IM was associated with higher remission rates, lower relapse rates, and comparable safety profiles. Larger RCTs should be conducted and focused on exploring the genetic and geographic differences between different cohorts.

## Introduction and background

Immunoglobin G4-related disease (IgG4-RD) is an immune-mediated systemic fibroinflammatory disease, that was first described in a Japanese cohort at the beginning of the 21st century [1]. It is characterized by enlargement of one or more organs, dense lymphoplasmacytic infiltrate rich in IgG4-positive plasma cells, and storiform fibrosis, with or without elevated serum IgG4 levels [2,3]. It commonly affects the biliary tract and the pancreas, lacrimal and salivary glands, kidneys, aorta, lungs, and the thyroid gland [4-6]. Diagnosis of IgG4-RD depends on several clinical, including localized or diffuse swelling, hypertrophy of, or nodular lesions in the affected organ, laboratory, radiological, and histopathological findings [7].

Multiple treatments for IgG4‐RD have been suggested, including surgery, systemic glucocorticoids (GC), immunosuppressive drugs (IM), and biologic agents [8,9]. GCs are the first line for treating IgG4-RD and most patients are sensitive to steroid therapy; however, relapses commonly occur during and after the treatment. Therefore, maintenance therapy is essential [8]. IM agents, such as cyclophosphamide (CTX), mycophenolate mofetil (MMF), and azathioprine (AZA) have been used for IgG4-RD. Nevertheless, controversy exists regarding the ideal timing of administration and its efficacy [9].

Previous individual studies are few and did not have sufficient power to reach statistical significance for the effectiveness of the combination of GC and IM compared to GC alone [10-13]. Further, no previous meta-analysis has been performed on the topic. Therefore, we conducted the current systematic review and meta-analysis to assess the safety and efficacy of GC monotherapy versus the combination of GC and IM for the treatment of patients with IgG4-RD.

## Review

Materials and methods

We followed the preferred reporting items of PRISMA statement guidelines, and meta-analysis of observational studies during drafting our manuscript [14,15]. Because this study was a systematic review and meta-analysis, formal ethical approval was not required.

Literature search strategy

We identified relevant studies by a systematic search of Medline via PubMed, Cochrane Central Register of Controlled Trials, Scopus, OVID, and Web of Science, till January 2021, using various combinations of the keywords: “IgG4-related disease”, “IgG4-RD”, “IgG4”, “glucocorticoids”, “steroid”, “cortisone”, “immunosuppression”, and “immunosuppressive agents”. No language restrictions were applied to the search. We manually checked the reference lists of identified articles.

Eligibility criteria and study selection

We included randomized controlled trials (RCTs) and observational studies that compared GC monotherapy versus the combination of GC and IM for IgG4 RD. We excluded animal models, reviews, case reports, case series, non-English articles, and duplicate references. We conducted eligibility screening in two steps: a) title and abstract screening to match the inclusion criteria, and b) full-text screening for eligibility to meta-analysis. Disagreements were resolved upon consensus.

Data extraction

Requisite data were extracted into a data extraction form. The extracted data included the following items: first author, year of publication, study design, country, mean follow-up, number of patients, sex, mean age of patients, interventions, dose of GC, types of IM, predominant organ involvement, IgG4-RD responder index (RI), risks of relapse, number of organs involved, duration of disease, blood tests (including eosinophils, erythrocyte sedimentation rate (ESR), c-reactive protein (CRP), and IgE), relapse rate, remission rate, and adverse events.

Risk of bias assessment

The quality of the retrieved RCTs was assessed according to the Cochrane Handbook of Systematic Reviews of Interventions [16]. The quality of the included studies was assessed by using the Newcastle Ottawa scale (NOS) [17]. Each included study was assessed based on reporting of three essential domains: a) selection of the study subjects, b) comparability of groups on demographic characteristics and important potential confounders, and c) ascertainment of the prespecified outcome (exposure/treatment).

Data synthesis

Included data (relapse, remission, and adverse events) were pooled as odds ratio (OR), with a 95% confidence interval (CI). We used R software (meta-package 4.9-4) for Windows during data synthesis.

Heterogeneity was assessed by visual inspection of the forest plots and measured by Q statistic and I2 statistic. Significant statistical heterogeneity was indicated by Q statistic P-value less than 0.1 or by I2 more than 50%. In case of significant heterogeneity, a random effect model was employed. Otherwise, the fixed effect model was used.

Results

Search Strategy Results

**Figure 1:**
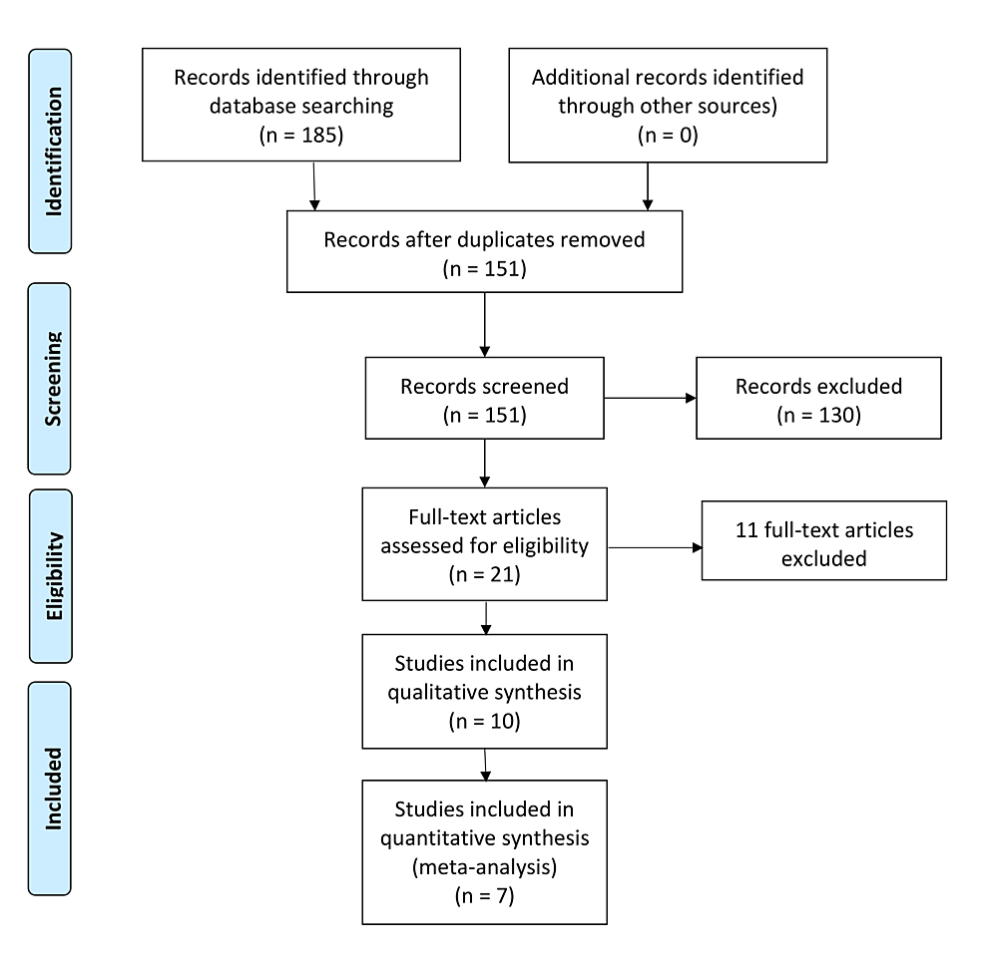
PRISMA flow diagram of study selection. This figure was drawn by the authors of this article.

Our literature search yielded 151 unique records. After title and abstract screening, 21 were retrieved and screened for eligibility. After full-text screening, 10 studies were included in the systematic review; of them, seven studies were available for meta-analysis. The flow of the study selection process is shown in Figure 1.

Characteristics of included studies

Ten studies (one RCT and nine observational studies) involving 906 patients were included [10-13,18-23]. Countries included in these studies were China (six studies), Italy (one study), the USA (one study), India (one study), and Singapore (one study). The follow-up period ranged from 3 to 36 months. All articles were published in English from 2014 to 2019. The included studies used several types of IM involving CTX, AZA, MMF, methotrexate (MTX), tripterysium glycosides, leflunomide (LEF), and cyclosporine A (CyA). Increased serum IgG4 and multiple organ involvement were the most common causes of relapse. Characteristics and summary of included studies are presented in Table 1.

**Table 1:** Summary table of the included studies. GC: glucocorticoid; IM: immunosuppressive agents; CTX: cyclophosphamide; AZA: azathioprine; MMF: mycophenolate mofetil; MTX: methotrexate; T2: tripterysium glycosides; LEF: leflunomide; CyA: cyclosporine A; NA: not available.

Author, year	Study design	Country	Mean follow-up (in months)	Number of patients (GC/GC+IM)	Sex, male (GC/GC+IM)	Mean age (years) (GC/GC+IM)	Treatment	Control	Dose of steroid	IM agents
Yunyun et al. 2019 [12]	Randomized clinical trial	China	12	35/34	25/20	55.34/56.76	GC	GC and IM	Started with 0.6 to 0.8 mg/kg/day, tapered at 5 mg, maintained at 410 mg/d	MMF, MTX, CYC, LEF
Hong et al. 2018 [11]	Retrospective study	China	24.6 (14.9)	81	28	51.8	GC	GC and IM	Started with 200 mg/d, followed by 0.6 mg/kg/day, taped by 2.5-5 mg, maintained at 5 mg/day	CTX, AZA, LEF, MMF
Wang et al. 2018 [10]	Prospective cohort study	China	22	77/138	52/96	54/54	GC	GC and IM	Started with 0.5 to 1.0 mg/kg, tapered by 5 mg, maintained by 5 to 10 mg/day	CTX, MMF, MTX, AZA, T2, LEF, CyA, CTX+LEF
Gupta et al. 2018 [20]	Retrospective study	India	3 to 28	58	12	44.1 (92.19)	GC	GC and IM	Started with 0.5 mg/kg/day to 1 mg/kg/day	AZA, MMF
Yunyun et al. 2017 [13]	Prospective cohort study	China	15 to 36	102	40/40	49.72/53.52	GC	GC and IM	Started with 0.5-1 mg/kg/d, maintained at 5-10 mg/d	CYC
Sekiguchi et al. 2016 [22]	Retrospective study	USA	29	166	125	61	GC	GC and IM	Started with 0.1 mg/kg	AZA, MMF
Campochiaro et al. 2016 [18]	Retrospective study	Italy	36	40	26	62 (55-67)	GC	GC and IM	Stated with 37.5 mg/day	AZA, MTX, MMF, CTX, RTX
Fong et al. 2018 [23]	Retrospective study	Singapore	4.1	42	31	66.3	GC	GC and IM	≤0.5mg/ kg, 0.51-0.99mg/kg, or ≥1.0mg/ Kg based on physician discretion	AZA, MMF, MTX
Lin et al. 2015 [21]	Prospective study	China	24	115	82	53.1 (13.6)	GC	GC and IM	NA	NA
Chen et al. 2014 [19]	Prospective study	China	6.3	28	18	51.5	GC	GC and IM	Started with 0.5–0.8 mg/kg/day), tapered to a maintenance dose of less than 10 mg/day	CTZ, AZA, MMF, MTX

Baseline data were collected and compared for age, sex, number of organs involved, duration of disease, IgG4-RD RI, and serology of patients (including eosinophils, ESR, CRP, IgE, and IgG4), and the results showed that there was no significant difference between the GC monotherapy group and the combination of GC and IM group for these baseline characteristics (Table 2).

**Table 2:** Baseline patient characteristics in studies comparing glucocorticoid monotherapy versus the combination of glucocorticoid and immunosuppressive agents in patients with IgG4-RD. CRP: C-reactive protein; ESR: erythrocyte sedimentation rate; IgG4: immunoglobulin G4-related disease; MD: mean difference; OR: odds ratio; CI: confidence interval.

	Number of studies reporting the variables	Number of patients included in each variable	Effect size comparing GC monotherapy versus GC and IM	P value	I^2^ value
Age	2	284	MD=-1.02 years, 95% CI [-2.23, 0.19]	0.1	0%
Male sex	3	386	OR=1.02, 95% CI [0.65, 1.60]	0.93	0%
Number of organs involved	2	171	MD=-0.13, 95% CI [-0.88, 0.61]	0.72	0%
Duration of disease	2	284	MD= 0.00 months, 95% CI [-4.93, 4.93]	1.00	0%
Eosinophils	3	386	MD=-0.01, 95% CI [-0.05, 0.03]	0.54	34%
CRP	3	386	MD=-0.31 mg/L, 95% CI [-1.37, 0.75]	0.57	54%
ESR	3	386	MD=-3.02 mm/h, 95% CI [-9.60, 3.56]	0.35	89%
IgE	3	386	MD= 22.34KU/L, 95% CI [-170.71, 215.38]	0.82	90%
IgG4	3	386	MD=-1286.76mg/dl, 95% CI [-5190, 56, 2617.04]	0.52	82%
IgG4-responder index	3	386	MD=-0.54, 95% CI [-1.15, 0.06]	0.08	0%

Risk of bias assessment

The included randomized clinical trial was at low risk of bias in terms of random sequence generation, incomplete outcome data, and selective reporting. While it was at high risk of bias regarding blinding. The included observational studies achieved a mean of 7 out of 9 points on the NOS, indicating a moderate quality of evidence. The summary of the risk of bias of both included trial and observational studies is presented in Appendices.

Summary of the results of the studies included in the systematic review

Three studies were included in the systematic review only because their data were insufficient for meta-analysis. Fong et al. involved 34 patients with IgG4-RD, who were managed with GC and combination IM agents. All patients responded to therapy by 3 months. After a median follow-up of 4.1 years, 25 patients needed a low dose of GC to maintain disease remission. About nine patients relapsed, of which seven patients had disease recurrence in the same organs [23]. Lin et al. conducted a prospective study on 116 patients with IgG4-RD treated with GC alone or in combination with IM drugs and found that the majority of patients improved within 3 months [21]. Chen et al. enrolled 28 patients with IgG4-RD. Most of the patients (n=26) were given prednisone and 19 patients were given IM agents. ESR and CRP were significantly decreased after treatment and serum IgG and IgG4 levels decreased over time [19].

Meta-analysis results

Relapse

**Figure 2:**
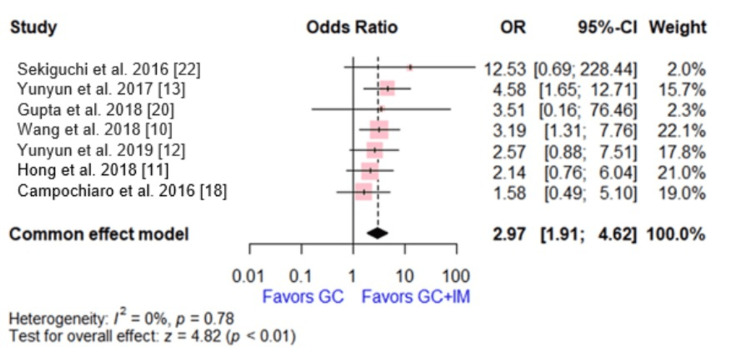
Forest plot comparing glucocorticoid versus glucocorticoid and immunosuppressive agents regarding relapse. This plot was drawn by the authors of this article.

Seven studies reported on relapse, with a total of 656 patients. The effect size showed that the GC group was associated with a higher relapse rate than the combination of GC and IM (OR = 2.97, 95% CI [1.91, 4.62], p < 0.0001). No significant heterogeneity was observed (p = 0.78, I2 = 0%), (Figure 2).

Complete Remission

**Figure 3:**
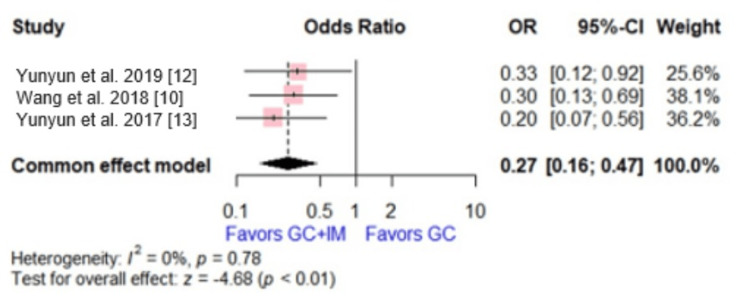
Forest plot comparing glucocorticoid versus glucocorticoid and immunosuppressive agents regarding complete remission. This plot was drawn by the authors of this article.

Three studies reported complete remission, with a total of 386 patients. The effect size showed that the GC group had a significantly less complete remission rate than the combination of GC and IM group (OR = 0.27, 95% CI [0.16, 0.47], p < 0.0001). No significant heterogeneity was observed (p = 0.78, I2 = 0%) (Figure 3).

Adverse Events

**Figure 4:**
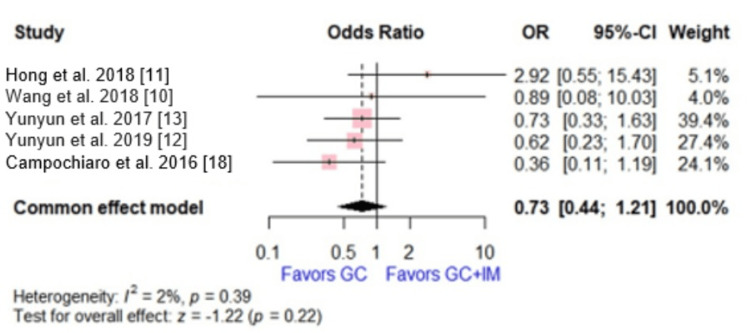
Forest plot comparing glucocorticoid versus glucocorticoid and immunosuppressive agents regarding adverse events. This plot was drawn by the authors of this article.

Five studies, involving 525 patients, reported adverse events (including glucose intolerance, newly diagnosed or aggravation of diabetes mellitus, infection, and any adverse events). The effect size showed no significant difference between the two compared groups (OR = 0.73, 95% CI [0.44, 1.21], p = 0.22). No significant heterogeneity was detected (p = 0.39, I2 = 3%) (Figure 4).

Subgroup Analysis According to Countries

Regarding relapse, the effect size from four Chinese studies favored GC+IM over GC monotherapy (OR = 3.04, 95% CI [1.85, 5.00], p <0.001). While the studies from the USA, Italy, and India did not favor either group (OR = 12.53, 95% CI [0.69, 228.44], p = 0.09, OR = 1.58, 95% CI [0.49, 5.10], p = 0.14, and OR = 3.51, 95% CI 0.16, 76.46], p = 0.43, respectively). Regarding complete remission, all included studies were from China; therefore, subgroup analysis was not applicable. Regarding adverse events, subgroup analysis of both Chinese and Italian studies held consistent with the overall estimate (OR = 0.85, 95% CI [0.49, 1.48], p = 0.51 and OR = 0.36, 95% CI [0.11, 1.19], p = 0.1, respectively).

Discussion

We performed this study because patients with IgG4-RD require long-term treatment to avoid relapse after achieving remission. Several GC-sparing agents have been suggested, but have been tested in small studies. This systematic review and meta-analysis compared the safety and efficacy of combination therapy of GC and IM vs. GC alone in the management of IgG4-RD. Our results showed that the combination therapy was associated with a higher rate of complete remission, lower relapse rate, and similar adverse events rate, compared to the GC alone group. In addition, the two groups were comparable in terms of age, sex, number of organs involved, duration of disease, IgG4-RD RI, and serology of patients (including eosinophils, ESR, CRP, IgE, and IgG4).

The improved efficacy after adding IM agents may be explained by their ability to disrupt the pathogenic mechanisms of IgG4-RD as T-cell activation, myofibroblast secretion of extra-cellular matrix, and production of pro-fibrotic cytokines [1]. Further, agents like MTX influence the function of memory T cells by inducing apoptosis in activated clones and disrupting the cross-talk between T lymphocytes and synovial fibroblasts [24].

This study helps to identify the published characteristics of patients with this rare disease. For example, the majority of published studies were conducted in East Asian countries (China [10,12,13,19,21], India [20], and Singapore [23]). The commonly prescribed IM agents included CTX, AZA, MMF, MTX, and LEF.

The subgroup analysis based on the country of origin may give some insights into the differences in disease characteristics and therapeutic responses in different ethnicities. For example, the studies from China showed a lower relapse rate in the combination group than in the GC group, while studies from other regions did not. Although this may be explained by the small sample size of patients from other regions, it may indicate different severities or modes of disease in different ethnicities. More research on the possible geographic and genetic factors that lead to these differences is encouraged.

More novel agents have been suggested for the management of patients with IgG4-related disease. For example, B-cell depletion with rituximab (a monoclonal antibody) has been tested before with promising results [25]. However, this treatment is costly and not available for many patients. Similar, more humanized monoclonal antibodies such as ocrelizumab may be considered as well. Therefore, the search for alternative GC-sparing agents continues.

The value of IgG4 serum concentration as an indicator of disease activity remains debated [26]. In our study, the risk of relapse was marked in some studies by elevated serum IgG4 levels, as well as multiorgan involvement, and increased eosinophilic count. While serum IgG4 levels may decrease with GC treatment, they might remain above the normal range at times, in spite of treatment [10,12].

Limitations

The IgG4-related disease is quite rare; therefore, the overall number of analyzed patients per outcome in this meta-analysis was relatively small (the biggest included study enrolled 166 patients). Many included studies were retrospective studies, which can give rise to incomplete data with possible recall bias. Future studies are encouraged to use larger sample sizes and employ a randomized controlled model to establish the evidence for this comparison.

## Conclusions

The treatment of IgG4-RD patients with a combination of GC and IM was associated with higher remission rates, lower relapse rates, and comparable safety profiles. Larger RCTs should be conducted and focused on exploring the genetic and geographic differences between different cohorts.

## Appendices

**Table 3:** Risk of bias assessment of included randomized clinical trial.

Yunyun et al. 2019 [12]	Risk of Bias	Reason/Quotations
Random sequence generation (selection bias)	Low risk	“The 69 patients were allocated by a random number generator”
Allocation concealment (selection bias)	Unclear	
Blinding of participants and personnel (performance bias)	High risk	Open label study
Blinding of outcome assessment (detection bias)	High risk	Open label study
Incomplete outcome data (attrition bias)	Low risk	The clinical data from all patients were used for intention to treat (ITT) analysis
Selective reporting (reporting bias)	Low risk	Protocol is not available but it is expected that all major outcomes were reported.
Other bias	Unclear	

**Table 4:** Methodological quality of the included observational studies based on the NOS for assessing the quality of epidemiological studies. ^a^If the exposure data was obtained from prescription database or medical record, a point was assigned. ^b^If the study design is prospective study, a point was assigned. ^c^If adjusted for age, a point was assigned. ^d^If adjusted for any other additional factors, a point was assigned. ^e^If the completeness of follow-up was 80% or more, a point was assigned. NOS: the Newcastle Ottawa scale​​​​​​​

Study	Selection				Comparability	Exposure			Total Score
	Representativeness of the exposed cohort	Selection of the non-exposed cohort	Ascertainment of exposure^a^	Outcome was not present at start of study^b^	Control for 2 important factors^c,d^	Assessment of outcome	Follow-up long enough	Adequacy of follow-up of cohort^e^	
Chen et al. 2014 [19]	*	*	*	*	*	*	-	*	7
Campochiaro et al. 2016 [18]	*	*	*	-	*	*	*	*	7
Lin et al. 2015 [21]	*	*	*	*	*	*	*	*	8
Sekiguchi et al. 2016 [22]	*	*	*	-	*	*	*	*	7
Gupta et al. 2018 [20]	*	*	*	-	*	*	*	*	7
Yunyun et al. 2017 [13]	*	*	*	*	*	*	*	*	8
Fong et al. 2018 [23]	*	*	*	-	*	*	-	*	6
Hong et al. 2018 [11]	*	*	*	-	*	*	*	*	7
Wang et al. 2018 [10]	*	*	*	*	**	*	*	*	9
